# The clinical characteristics of corticosteroid-resistant refractory *Mycoplasma Pneumoniae* pneumonia in children

**DOI:** 10.1038/srep39929

**Published:** 2016-12-23

**Authors:** Yongdong Yan, Yuzhen Wei, Wujun Jiang, Chuangli Hao

**Affiliations:** 1 Department of Respiratory Medicine, Children’s Hospital of Soochow University, Soochow University, Suzhou, China; 2 Department of Pediatrics, XuZhou Children’s Hospital, Xuzhou, China

## Abstract

To analyze the clinical characteristics of corticosteroid-resistant refractory *Mycoplasma pneumoniae* pneumonia (RMPP) and explore the related factors that predict corticosteroid-resistant RMPP. Retrospective analysis of 183 children with RMPP in our hospital admitted between January 1, 2012, and December 31, 2014 was performed. Of the 183 RMPP cases, 36 (19.7%) were corticosteroid-resistant RMPP cases. Corticosteroid-resistant RMPP cases had a longer duration of fever and hospitalization compared with corticosteroid-sensitive RMPP cases (P < 0.05). The radiographic findings of 123 (83.7%) cases of corticosteroid-sensitive RMPP apparently resolved after one week of corticosteroid treatment compared with 4 (11.1%) corticosteroid-resistant RMPP cases that had apparently resolution (P < 0.01). Twenty-four (75%) corticosteroid-resistant RMPP patients who received bronchoscopy had mucus plug formation while none of the corticosteroid-sensitive RMPP patients had mucus plug formation (P < 0.05). Multiple logistic regression analysis showed that duration of fever ≥11 days, percentage of lymphocytes ≤32%, CRP ≥48.73 mg/L and LDH ≥ 545.7 U/L were significant predictors of corticosteroid-resistant RMPP. Patients with corticosteroid-resistant RMPP had more severe presentations and more serious radiological findings. Clinicians might use the parameters of duration of fever, CRP, LDH and proportion of lymphocytes to identify children at higher risk of corticosteroid-resistant RMPP.

*Mycoplasma pneumoniae* is one of the most important pathogens in children and young adults, accounting for 10–40% of community-acquired pneumonia^1^,^2^,^3^,^4^. It has been observed in many areas of the world that epidemics of *M. pneumoniae* infections occur every 3–7 years^5^,^6^,^7^, which increases the rate of morbidity, mortality, as well as the cost of health care in society. Although mycoplasmal pneumonia is usually self-limited and benign, some cases may proceed to clinical and radiological deterioration despite appropriate macrolide therapy, which were defined as refractory *M. pneumoniae* pneumonia (RMPP). The rapidity of response to treatment of RMPP with systemic corticosteroid is satisfying, and it significantly improves the clinical symptoms and outcomes^8^,^9^. Nonetheless, with the use of standard corticosteroid regimens (2 mg/kg/day), fever persists for more than 3 days after treatment in around 20% of children with RMPP^10^.

We encountered several severe cases of RMPP that were unresponsive to standard doses of corticosteroid and required investigation using fiber optic bronchoscopy. Therefore, we were interested in our institution’s experience of RMPP patients who were unresponsive to corticosteroids. We retrospectively analyzed all children with RMPP over a 3-year period to determine the clinical features, laboratory data, radiological findings and fiber optic bronchoscopic findings of these patients who failure to fully respond to corticosteroid and report our experience of treating these children.

## Results

### Clinical characteristics

**Figure 1:**
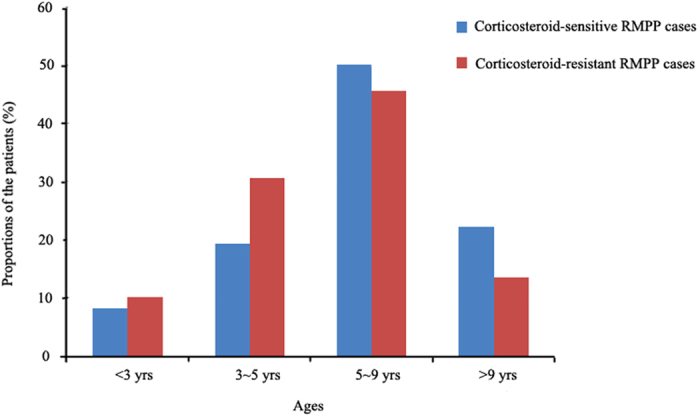
The age distribution of patients with corticosteroid-resistant and corticosteroid-sensitive refractory M. pneumoniae pneumonia.

A total of 183 RMPP cases were analyzed in the present study consisting of 147 (80.3%) corticosteroid-sensitive cases and 36 (19.7%) corticosteroid-resistant cases. The mean age of the patients was 6.20 ± 2.59 years with a male to female ratio of 1.08. Most (61.7%) of the RMPP patients were older than 5 years old, and the age group of 5–9 years had the highest proportion of both corticosteroid-resistant and corticosteroid-sensitive patients. The age distribution is shown in Fig. 1. No difference was found in the male/female ratio, age, duration of fever before hospitalization, and occurrence of fever, cough and wheezing between the two groups. However, corticosteroid-resistant RMPP cases had a longer duration of hospitalization compared with corticosteroid-sensitive RMPP cases. Moreover, they had higher incidences of tachypnea and rales (all P < 0.05). The results are shown in Table 1.

### Laboratory findings

The laboratory values of the corticosteroid-resistant and corticosteroid-sensitive RMPP cases at admission are shown in Table 2. WBC, N%, LDH, CRP, and the number of CD3 − CD19+ cells and CD19 + CD23+ cells were higher in the corticosteroid-resistant RMPP cases. On the other hand, L%, PLT, and number of CD3+ cells and CD3 + CD4+ cells were higher in the corticosteroid-sensitive RMPP cases.

### Radiographic findings

Pleural effusion was observed in 28 (19.0%) of the corticosteroid-sensitive RMPP cases and in 17 (47.2%) of the corticosteroid-resistant RMPP cases; the difference was statistically significant (P < 0.01). However, there was no difference in the incidence of atelectasis between the two groups (12.9% vs. 11.1%, P > 0.05). The radiographic findings of 123 (83.7%) cases of corticosteroid-sensitive RMPP apparently resolved after one week of corticosteroid treatment compared with 4 (11.1%) corticosteroid-resistant RMPP cases that had resolution (P < 0.01).

### Treatment

Concerning the course of treatment, we found that the corticosteroid-resistant patients had a longer corticosteroid treatment course than the corticosteroid-sensitive patients (12.33 ± 3.56 days vs. 7.25 ± 1.78 days, respectively, P < 0.01), and such a difference was also found in the treatment course of non-macrolide antibiotics (11.33 ± 3.01 days vs. 8.37 ± 2.51 days, respectively, P < 0.01). However, there was no difference in the initial treatment duration for azithromycin (P > 0.05). Twenty-one of the 36 (58.3%) corticosteroid-resistant RMPP cases defervesced within 120 hours after intravenous pulse methylprednisolone of 2 mg/kg/day. Intravenous pulse methylprednisolone of 4 mg/kg/d was administered on the 3^rd^ day in the remaining 15 cases and as a result, 7 cases defervesced within 48 hours. In the remaining two cases, one defervesced after 400 mg of intravenous IVIG for two consecutive days and the other defervesced within 24 hours after intravenous pulse methylprednisolone of 6 mg/kg/day on the 4^th^ day.

### Bronchoscopy intervention

**Figure 2:**
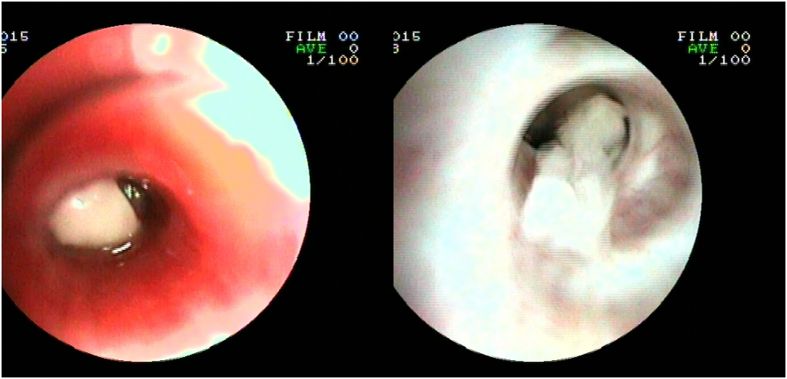
Bronchoscopic findings of the patient with mucus plug formation.

A total of 57 RMPP patients received bronchoscopy (24 [16.4%] in the corticosteroid-sensitive group vs. 32 [88.9%] in the corticosteroid-resistant group, P < 0.01). Twenty-four (75%) corticosteroid-resistant patients who received bronchoscopy had mucus plug formation (Fig. 2), while none of the corticosteroid-sensitive patients who received bronchoscopy had mucus plug formation.

We further compared the clinical characteristics of corticosteroid-resistant RMPP patients with mucus plug formation (n = 24) and those without mucus plug formation (n = 8). The mean age of mucus plug and non-mucus plug patients was 6.3 ± 3.0 years and 5.3 ± 3.2 years, respectively, with a significant difference (P < 0.05). Male percentage was 58.3% (14/24) in the mucus plug patients and 62.5% (5/8) in the non-mucus plug patients with no statistical difference. The mucus plug patients had a longer median (range) fever duration (12 [11, 21] vs 10 [9, 12], P < 0.05) and a longer hospital stay (14 [12, 24] vs 11[6, 15], P < 0.05).

### Risk factors for corticosteroid-resistant RMPP

Univariate analysis identified duration of fever, peak body temperature, pleural effusion, rash, WBC, N%, L%, PLT, LDH, CRP, CD3+ cells, CD3 + CD4+ cells, CD3 − CD19+ cells and CD19 + CD23+ cells as significant corticosteroid-resistant RMPP risk factors. Multivariate logistic regression identified duration of fever, L%, CRP and LDH as independent risk factors for corticosteroid-resistant RMPP after adjustment for confounders (P < 0.05, Table 3). The cut-off values for duration of fever, L%, CRP and LDH were 11 days, 32%, 48.73 mg/L and 545.7 U/L, respectively.

## Discussion

In the present study, we retrospectively reviewed and analyzed the medical data of RMPP patients and found that corticosteroid-resistant patients had a longer duration of fever, length of hospitalization, and higher incidence of tachypnea, rales and rash. They also had a higher incidence of pulmonary complications such as pleural effusion. Regarding laboratory values, significant differences between corticosteroid-resistant and corticosteroid-sensitive RMPP cases in WBC, N%, LDH, CRP, L%, PLT and some subtypes of T cells were observed. Moreover, we identified a longer duration of fever, higher levels of CRP and LDH, and lower L% as risk factors associated with resistance of RMPP to corticosteroids. As far as we know, corticosteroid-resistant RMPP has not been studied systematically before and this is the first study focused on the clinical characteristics and laboratory values, as well as radiographic findings.

Corticosteroid has been used for RMPP and had a promising efficacy, though its underlying mechanisms are uncertain. Host cell-mediated immunity plays an important role in the development of the pulmonary lesions caused by *M. pneumoniae* and this was defined by an examination in immunocompromised hosts^11^. In our study, we found higher numbers of CD3 − CD19+ and CD19 + CD23+ and lower levels of CD3+ and CD3 + CD4+ cells in the corticosteroid-resistant RMPP cases. The excessive inflammation reaction may lead to the immune disorder, which might be related to the severity of RMPP in children. Previous studies revealed that strong cellular immunological responses cause severe cilia abnormalities in RMPP^12^,^13^. Severe cilia abnormalities reduce the immune function of the airways and disrupt mucociliary clearance, thereby causing the mucous plugs that are responsible for the development of large infiltrations or atelectasis on chest radiographs. In our study, 24 (75%) corticosteroid-resistant patients who received bronchoscopy had mucus plug formation, while none of the corticosteroid-sensitive patients who received bronchoscopy had mucus plug formation, suggesting that an excessive inflammation reaction in corticosteroid-resistant patients is associated with the formation of mucus plugs.

To investigate the risk factors for corticosteroid resistance, we chose variables that are commonly examined in our hospital together with demographic and clinical characteristics. Four independent factors of duration of fever, CRP, LDH and L% were identified. LDH was a variable that is universally elevated in many pulmonary diseases and was reported to be associated with disease severity in several studies^14^,^15^. Previous studies had identified higher levels of LDH as a risk factor for RMPP^16^,^17^,^18^. CRP is a gross biochemical index of inflammation and is used commonly in the clinical setting. CRP reflected the severity of acute systemic inflammatory reactions to *M. pneumoniae* infection. Zhang *et al*. found that CRP ≥ 16.5 mg/L was a significant predictor of RMPP^18^. In our study, we found that in RMPP patients, higher LDH and CRP were also biomarkers for predicting corticosteroid-resistant RMPP. The severity of *M. pneumoniae* infection tended to be inversely associated with lymphocyte counts in children^19^. We also found that lymphopenia may be one of the characteristics of corticosteroid-resistant RMPP.

Our study had some limitations. First, as this was a retrospective study, select bias might exist and further prospective studies are potentially needed. Second, the patients are from the same province, so the risk factors associated with corticosteroid resistance might not be applicable to patients in other areas, and a multi-center study is needed in the future. Third, there might be some cases that were co-infected with other pathogens that could not be detected and might therefore result in both corticosteroid-resistant and corticosteroid-sensitive RMPP.

In conclusion, patients with corticosteroid-resistant RMPP presented with more severe presentations and had more serious radiological findings. Clinicians might use duration of fever, CRP, LDH and L% for identifying children at a higher risk of corticosteroid-resistant RMPP.

## Methods

### Study patients

All experiments were performed following the relevant guidelines and regulations of Soochow University. The methods were carried out in accordance with the approved guidelines. The study was approved by the Medical Ethics Committee of Soochow University. The parents of all study participants gave both verbal informed consent before study enrollment. We retrospectively collected the data of patients with RMPP who were admitted to the Department of Respiratory Medicine in the Children’s Hospital of Soochow University between January 1, 2012 and December 31, 2014. The exclusion criteria for our study were (1) patients with congenital heart diseases, heredity metabolic diseases, neurological disorders, bronchopulmonary dysplasia, and immunodeficiency; (2) those co-infected with other pathogens; (3) those with incomplete clinical data; and (4) those in the convalescent stage of the disease.

### Definitions

*M. pneumoniae* pneumonia was confirmed when (1) a pulmonary infiltrate on a chest radiograph was present in combination with fever, cough or auscultatory findings that were consistent with pneumonia; and (2) A significant rise in *M. pneumoniae* IgG or seroconversion in paired sera, or the presence of IgM antibodies together with *M. pneumoniae* DNA. RMPP were defined as cases showing clinical and radiological deterioration despite appropriate antibiotic therapy for 7 days or more^20^. Corticosteroid-sensitive RMPP was defined as defervescence by 72 hours after the regimen of intravenous methylprednisolone of 2 mg/kg/day and no return of fever for at least 7 days after corticosteroid. Corticosteroid-resistant RMPP had persistent or recrudescent fever >72 hours after the regimen of intravenous methylprednisolone of 2 mg/kg/day. Fiber optic bronchoscopy was indicated when radiographic findings showed lobar/segmental consolidation or atelectasis after appropriate antibiotic and corticosteroid treatment for 1 week.

### Data collection

Demographic, clinical information, laboratory data, radiological and fiber optic bronchoscopic findings were retrospectively collected from the records of all children. White blood cells (WBC), proportion of neutrophils (N %), proportion of lymphocytes (L %), platelet count (PLT), lactate dehydrogenase (LDH), alanine transaminase (ALT), IgA, IgG, IgM, and cell-mediated immunity were analyzed on admission. Nasopharyngeal aspirates were routinely collected within 24 hours of admission. Microbiologic tests were performed to exclude other respiratory tract infections, including blood cultures, nasopharyngeal aspirates for common respiratory tract virus antigens (respiratory syncytial virus, influenza virus A, influenza virus B, parainfluenza virus 1, parainfluenza virus 2, parainfluenza virus 3, and adenovirus). All patients were administered azithromycin (10 mg/kg/day) together with or without a broad-spectrum antibiotic.

### Real-time PCR for *M. pneumoniae* detection

Nasopharyngeal swabs were obtained within 24 h of admission. The specimens were centrifuged and were stored at −80 °C. A quantitative diagnostic kit (DaAn Gene Co., Ltd. Guangzhou, China) for *M. pneumoniae* DNA was used to measure the load of *M. pneumoniae*. The method is based on TaqMan PCR technology, and the target is 16S rRNA gene specific for *M. pneumoniae* genome. Briefly, 1 mL of nasopharyngeal aspirates diluted by 4% NaOH was centrifuged at 12,000 rpm for 5 min. The sediment was collected, washed twice with 0.9% NaCl, blended with 50 μL of DNA extraction solution, incubated at 100 °C for 10 min, and centrifuged at 12,000 rpm for 5 min. Real-time PCR was performed on the resulting supernatant of 2 μL with 43 μL of PCR mix (supplied with the kits) using the DA 7600 real-time PCR system (Applied Biosystems, CA, USA) as follows: 93 °C for 2 min, 10 cycles of 93 °C for 45 s and 55 °C for 60 s, followed by 30 cycles of 93 °C for 30 s and 55 °C for 45 s. All nasopharyngeal swabs were tested by immunofluorescent antigen detection to identify seven common viruses (respiratory syncytial virus, adenovirus, influenza viruses A and B, and parainfluenza viruses 1, 2 and 3).

### M. pneumoniae serology

Specific IgM and IgG antibodies against *M. pneumoniae* were detected in serum samples of patients in the acute phase of *M. pneumoniae* pneumonia (on admission) and in the convalescent phase (on discharge), respectively, using a commercial ELISA kit (Serion ELISA classic MP IgG/IgM, Institute Virion/Serion, Würzburg, Germany) according to the manufacturer’s instructions. The test cut-off value was 0.5 × mean optical density (OD) of the kit control serum, as indicated in the insert. A positive IgG reaction was defined as >24 RU/mL. A significant rise in IgG titre was considered to be a doubling of the OD value above the cut-off, or a sero-conversion in which the primary serum was antibody negative and the second serum had an OD at least twice the cut-off corresponding to a threefold rise in RU/mL titre. A positive IgM antibody reaction was defined as >1.1 S/CO.

### Statistical analysis

Statistical analyses were conducted using SPSS 22.0. Data are expressed as mean ± standard deviation (SD), median with range or number with percentage as appropriate. Parametric and nonparametric comparative tests for continuous data and χ2 test for categorical data were used to compare variables between groups. P < 0.05 was considered statistically significant. Multivariate logistic regression analysis was performed to analyze independent risk factors for corticosteroid-resistant RMPP selected by univariate analysis. To explore the predictive values of laboratory data for corticosteroid-resistant RMPP, receiver operator characteristic (ROC) curves were made and the cut-off values with maximum sensitivities and specificities were determined.

## Additional Information

**How to cite this article**: Yan, Y. *et al*. The clinical characteristics of corticosteroid-resistant refractory *Mycoplasma Pneumoniae* pneumonia in children. *Sci. Rep.* **6**, 39929; doi: 10.1038/srep39929 (2016).

**Publisher's note:** Springer Nature remains neutral with regard to jurisdictional claims in published maps and institutional affiliations.

## Figures and Tables

**Table 1:** Clinical characteristics of corticosteroid-resistant and corticosteroid-sensitive RMPP cases.

	Corticosteroid-sensitive (n = 147)	Corticosteroid-resistant (n = 36)	P value
Age, mean ± SD, years	6.11 ± 2.59	6.58 ± 2.63	0.65
Male/female ratio	78/69	17/19	0.53
Length of fever before hospitalization, median (ranges), days	7 (3, 14)	7 (3, 17)	0.77
Total length of fever, median (ranges), days	8 (5, 16)	12 (9, 21)	<0.01
Length of hospitalization, median (ranges), days	9 (5, 20)	13 (6, 24)	<0.01
Fever, n (%)	147 (100)	36 (100)	1.00
Cough, n (%)	147 (100)	36 (100)	1.00
Tachypnea, n (%)	5 (3.4)	15 (41.7)	<0.001
Wheezing, n (%)	31 (21.2)	9 (25.0)	0.61
Rales, n (%)	79 (53.7)	30 (83.3)	0.001
Decreased lung sounds, n (%)	69 (46.9)	18 (50.0)	0.74
Rash, n (%)	6 (4.1)	9 (25.0)	<0.001
Hepatic dysfunction, n (%)	16 (10.9)	3 (8.3)	1.00

RMPP: refractory *Mycoplasma pneumoniae* pneumonia; SD: standard deviation.

Hepatic dysfunction was defined as alanine transaminase >80 U/L.

**Table 2:** Laboratory values of the corticosteroid-resistant and corticosteroid-sensitive RMPP cases.

	Corticosteroid-sensitive (n = 147)	Corticosteroid-resistant (n = 36)	P value
WBC, mean ± SD, ×10^9^/L	7.44 ± 2.76	8.61 ± 3.65	<0.05
N%, mean ± SD	64.04 ± 11.33	74.80 ± 10.11	<0.01
L%, mean ± SD	26.74 ± 9.75	18.23 ± 8.33	<0.01
PLT, mean ± SD, ×10^9^/L	278.58 ± 87.76	246.17 ± 83.75	<0.05
LDH, mean ± SD, U/L	423.59 ± 135.05	674.13 ± 324.54	<0.01
CRP, mean ± SD, mg/L	25.30 ± 28.33	73.80 ± 49.04	<0.01
Cell-mediated immunity, %			
CD3+ cells	65.17 ± 8.94	58.31 ± 10.00	<0.01
CD3 + CD4+ cells	35.13 ± 7.38	31.23 ± 9.74	<0.01
CD3 + CD8+ cells	25.13 ± 6.18	23.24 ± 3.51	>0.05
CD3 − CD19+ cells	20.01 ± 7.46	25.40 ± 9.46	<0.01
CD3 − CD(16+56)+ natural killer cells	12.68 ± 7.77	14.37 ± 8.28	>0.05
CD19 + CD23+ cells	6.69 ± 3.73	8.19 ± 4.19	<0.05
Humoral immunity			
IgA, mean ± SD, g/L	1.26 ± 0.67	1.10 ± 0.59	>0.05
IgG, mean ± SD, g/L	8.04 ± 2.80	8.01 ± 2.02	>0.05
IgM, mean ± SD, g/L	1.62 ± 0.75	1.47 ± 0.57	>0.05

All the values are expressed as mean ± SD.

**Table 3:** Independent risk factors of corticosteroid-resistant RMPP.

Variables	OR	95% CI	P
Fever duration ≥11d	6.06	1.56~23.50	<0.01
L% ≤ 32%	0.90	0.85~0.94	<0.01
LDH ≥545.7U/L	1.01	1.00~1.03	<0.05
CRP ≥48.73 mg/L	1.05	1.01~1.09	<0.05

LDH: lactate dehydrogenase; CRP: C-reactive protein.
